# Three-Dimensional Structures of Carbohydrates and Where to Find Them

**DOI:** 10.3390/ijms21207702

**Published:** 2020-10-18

**Authors:** Sofya I. Scherbinina, Philip V. Toukach

**Affiliations:** 1 N.D. Zelinsky Institute of Organic Chemistry, Russian Academy of Science, Leninsky prospect 47, 119991 Moscow, Russia; 2 Higher Chemical College, D. Mendeleev University of Chemical Technology of Russia, Miusskaya Square 9, 125047 Moscow, Russia

**Keywords:** carbohydrate, spatial structure, model build, database, web-tool, glycoinformatics, structure validation, PDB glycans, structure visualization, molecular modeling

## Abstract

Analysis and systematization of accumulated data on carbohydrate structural diversity is a subject of great interest for structural glycobiology. Despite being a challenging task, development of computational methods for efficient treatment and management of spatial (3D) structural features of carbohydrates breaks new ground in modern glycoscience. This review is dedicated to approaches of chemo- and glyco-informatics towards 3D structural data generation, deposition and processing in regard to carbohydrates and their derivatives. Databases, molecular modeling and experimental data validation services, and structure visualization facilities developed for last five years are reviewed.

## 1. Introduction

Knowledge of carbohydrate spatial (3D) structure is crucial for investigation of glycoconjugate biological activity [1,2], vaccine development [3,4], estimation of ligand-receptor interaction energy [5,6,7] studies of conformational mobility of macromolecules [8], drug design [9], studies of cell wall construction aspects [10], glycosylation processes [11], and many other aspects of carbohydrate chemistry and biology. Therefore, providing information support for carbohydrate 3D structure is vital for the development of modern glycomics and glycoproteomics.

As result of growing interest to glycoprofiling, glycan microarrays, carbohydrate active enzymes (CAZy) and glycan-binding proteins (GBP) which are involved in biological processes, several major international projects (e.g., GlySpace [12], GlyCosmos [13], Glycomics@ExPASy [14], GlyGen [15], JCGGDB [16], Glytoucan [17], MIRAGE [18], CFG [19], RINGS [20], GLIC (https://glic.glycoinfo.org/), SysGlyco (https://sysglyco.org/)) were launched to integrate variety of data produced by glycobiological research. The main goal of existing glycoinformatics projects is to provide versatile resources with user-friendly access helpful for disease diagnostics [21,22], glycobioinformatics studies [23], glycosylation site prediction [24], CAZy activity prognosis [25,26] and other applications.

Appending of structural repositories with 3D structural data opens the way for computational glycobiology and modeling of carbohydrate structures at atomic resolution. Design of novel workflows and techniques to connect carbohydrate spatial structure modes and experimental data with verification, processing, analysis and deposition of associated data has gained increased popularity in glycoscience community [27]. A Carbohydrate Structure Database (CSDB, [28]) module for carbohydrate 3D structure modeling is a demonstrative example of 3D structural data integration facilities (as a database) combined with dedicated interface (as a glycoinformatics project). Further details on CSDB 3D facilities are discussed below.

**Figure 1:**
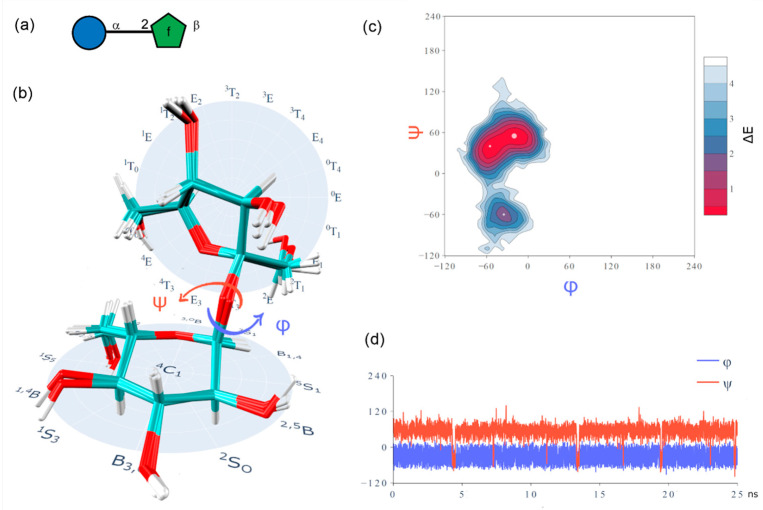
Typical components of a carbohydrate 3D structure exemplified on sucrose: (**a**) primary structure (in Symbol Nomenclature for Glycans (SNFG)); (**b**) superimposed conformational states and Cremer–Pople diagram; (**c**) conformational space of a two-torsion glycosidic linkage (Ramachandran plot); (**d**) transitions of glycosidic dihedrals.

The typical types of knowledge about a carbohydrate 3D structure include (Figure 1):

- Primary structure (atom connectivity);
- Monosaccharide ring conformation;
- Rotational states of inter-residue and exocyclic linkages and their energies;
- Ring puckering and transitions of glycosidic linkage conformation on a time scale;
- Large-scale spatial arrangement (tertiary structure).

Herein we focus on the important aspects of carbohydrate 3D structure availability to researchers: structural repositories; glycoinformatics tools and workflows to assist structure building, modeling and erroneous molecular geometry data detection and remediation; carbohydrate 3D structure presentation and visualization methods.

## 2. Structural Databases

**Figure 2:**
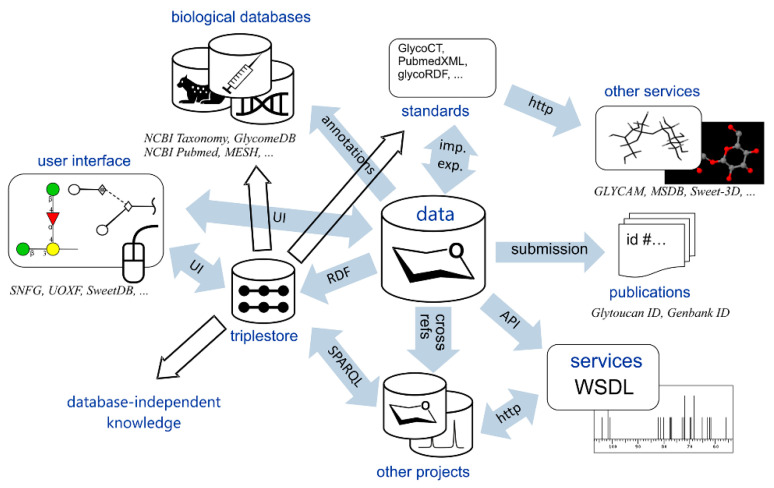
Networking between glycoinformatics projects and related services that promotes achievement of data integration in glycomics. Reproduced with permission from [29], © 2020 Wiley-VCH Verlag GmbH & Co. KGaA, Weinheim.

Structural databases make significant contribution to bringing information technologies to glycoscience [29]. With no focus on spatial structure, glycan databases and online tools have been recently reviewed [30,31,32]. Depositing huge number of carbohydrates with detailed data for each entry, databases are valuable sources of structural information, biological assignments, references and external links. Structural data are often accompanied by original and sometimes assigned experimental observables: NMR spectra, HPLC and MS profiles, etc. The services built on top of the databases can include 3D structure simulation, validation, and storage. A viewpoint of the authors at the ideal integration of data resources and services in glycoinformatics is summarized in Figure 2. A subject of this review is databases providing theoretical or empirical 3D structures of carbohydrates and related data-mining tools.

The majority of existing repositories for carbohydrate 3D structures offer open-access data via web interface. Deposited datasets can be represented by glycoproteins, protein-carbohydrate complexes, poly- and oligosaccharides with 3D structure experimentally resolved or specified by means of NMR, X-ray crystallography, cryoEM, small angle X-ray scattering, etc. [27]. Several databases such as GLYCAM-Web, EK3D, 3DSDSCAR, GlycoMapsDB contain data from molecular dynamics simulations. We have also mentioned databases featuring information on protein structures involving carbohydrate moiety in terms of glycosylation (as post-translational modification, dbPTM), carbohydrate active enzymes (CAZy) and homology modeling (SWISS-MODEL). Table 1 displays currently active structural databases maintaining three-dimensional data on carbohydrates.

For Table 1, we have selected carbohydrate and related databases using the following criteria:

- Database can be freely accessed through web user interface;
- Database must contain experimentally confirmed and/or predicted 3D structures (preprocessed and/or generated on-the-fly from a primary structure input) of glycans, glycoproteins, or protein-carbohydrate complexes;
- Stored 3D structures must be deposited as atomic coordinates in PDB, MOL, or other format, and the structures must contain a saccharide moiety;
- Databases with records linked to other large 3D data collections (e.g., RCSB PDB, PDBe, PDBj, PDBsum, UniProtKB etc.) are included in Table 1 (as long as database entries contain carbohydrate moiety, e.g., as a part of a lectin or an antibody);
- Databases with derived carbohydrate 3D structural data (conformational maps, conformer energy minima, etc.) are included in Table 1 even if they provide no atomic coordinates (e.g., GlycoMapsDB and GFDB).

Despite no fit to the criteria above, assistance of large structure repositories offering only glycan primary structures (e.g., GlyToucan [17] (https://glytoucan.org/), UniCarbKB [33] (http://www.unicarbkb.org/)) can be useful for cross-referencing of existing carbohydrate resources and serve as supplementation to 3D modeling pipelines.

Some out-of-date projects, such as Complex Carbohydrate Structural Database (CCSD) [34,35], EUROCarbDB [33,36], GlycomeDB [36,37,38], Glycoconjugate Data Bank [39], GlycoSuite [40,41] are noteworthy as they had shaped the modern vision of structural glycoinformatics.

## 3. Carbohydrate 3D Structure Modeling

Methods to probe a 3D structure of carbohydrate-containing biomolecules has been developed for decades. NMR techniques (interatomic distances derived from NOE, and torsion angles derived from coupling constants), X-ray crystallography, and electron cryo-microscopy (the two latter being atomic models built on the basis of electron density map) are among most demanded methods for 3D strucural elucidation. These methods have been reviewed [93,94,95,96] and are beyond the scope of this review focused in information technologies. For use of instrumental methods for the validation of a simulated structure, please refer to Section 5 “Experimental data validation”.

Structural investigation of large biological systems involving protein-glycan interactions requires leveraging more resources and employing more complex experimental techniques compared to solely oligo- and polysaccharides studies. Advances in NMR methods hold great potential for direct spatial structure determination of carbohydrate-protein complexes in solution based on intermolecular NOEs which affords estimation of atomic contacts between a protein and a carbohydrate ligand [97,98]. Further extraction of NOE-derived distance restraints for a saccharide molecule results in generation of representative conformational ensembles [99,100,101].

Support of experimental data with computer simulations can significantly improve quality of 3D structures. Quantum mechanics [100,102,103,104,105,106] and molecular dynamics modeling [107,108,109,110,111] are commonly applied to conformation search and NMR signal prediction.

To date, the following theoretical models and methods are applied for in silico design of carbohydrate three-dimensional structure [112,113,114,115,116]:

- Molecular mechanics (MM) and molecular dynamics (MD) calculations [117];
- Monte Carlo simulations [118,119];
- Semi-empirical methods [120,121,122,123];
- *Ab initio* simulations based on density functional theory (DFT) [124,125,126,127,128];
- Hybrid QM/MM and QM/QM and ONIOM (“our own N-layered integrated molecular orbital and molecular mechanics”) approaches [129,130,131,132,133,134].

Due to computational limitations, most of publications of the recent decade have reported molecular dynamics approaches in general or dedicated force fields. With increasing computer power, other methods gain interest, however majority of applications of molecular modeling of complex carbohydrates, especially in solution, still use MM/MD methods.

Based on Scopus [135] article count we estimated the application rate for quantum mechanics (10759 publications) and molecular mechanics (14871 publications) methods applied for carbohydrate structure modeling for the recent five years (2015–2020). Search queries included abundant carbohydrate terms, typical glycan moieties, and common modeling approaches (query details are given in Supplementary Table S1). In spite of growing interest to QM approaches in carbohydrate structure simulation, the major contribution to the statistics for such resource-intensive calculations is application of QM to relatively simple model compounds. For complex bioglycans in solution predominance of MM methods is more pronounced [6,8].

### Molecular Mechanics and Dynamics

Molecular dynamics methods have achieved broad scope of application in terms of reasonable computer resource consumption. They fulfill advantageous compromise between calculation accuracy and performance, when applied to glycan molecules and their structural complexity (variety of known monomeric elements, presence of ionogenic groups), high bridge flexibility and stereo-electronic effects [112,113,136,137].

In molecular mechanics simulations, Newtonian mechanics principles are applied to calculate potential energy of a system using parameter set specific for a class of compounds under study (force field). Particular features of carbohydrate moiety, e.g., ring puckering, rotational barriers, hydrogen bonds, must be taken into account to perform precise analysis of molecular behavior *in vacuo* or in solution [138].

**Figure 3:**
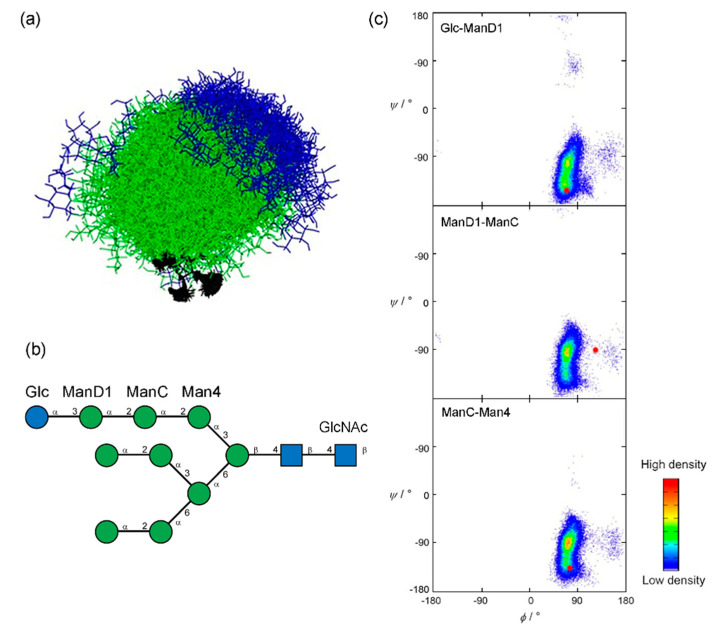
NMR-validated conformational analysis of high-mannose oligosaccharide GM9 based on replica-exchange molecular dynamics (REMD) simulation results. (**a**) Superimposition of 260 GM9 conformers extracted from REMD trajectory (black—GlcNAc, green—Man, blue—Glc). (**b**) primary structure of the GM9 oligosaccharide (SNFG representation). (**c**) REMD density maps for φ-ψ torsions of GM9 branch (Glc_1_Man_3_). Red dots locate glycosidic torsion angles derived from crystallographic data of Glc_1_Man_3_ tetrasaccharide ligand complexed with the lectin domain of calreticulin (PDB ID: 3O0W). Panels (**a**) and (**c**) were reproduced with permission from [149], © 2020 Wiley-VCH Verlag GmbH & Co. KGaA, Weinheim.

Molecular dynamics simulations consider Newtonian motion equations to observe evolution of a system during a certain timespan. Conformation ensemble generation occurs via calculation of molecular trajectory at given temperature. Accuracy of calculation depends on the employed force field and sufficient conformational sampling. MD simulations are commonly used for interpretation and analysis of the NMR and X-ray observables in the context of carbohydrate 3D structure [139]. Enhanced molecular dynamics sampling technologies, such as replica-exchange MD (REMD) [140,141], Hamiltonian replica-exchange MD (HREX) [142,143,144], multidimensional swarm-enhanced sampling MD (msesMD) [145,146], Gaussian accelerated MD (GAMD) [147,148] have been reported. Density maps or energy maps built for a set of the glycosidic torsion angles (φ, ψ, ω) are a typical way to report conformational preferences of a glycan provided by population analysis of its MD trajectory. As a representative example, conformational characteristics of highly flexible branched oligosaccharide Glc_1_Man_9_GlcNAc_2_ (GM9) were investigated by explicit-water REMD study and validated using paramagnetism-assisted NMR spectroscopy [149] (Figure 3a,b). Due to the structural complexity of GM9, adequate exploration of conformational space requires long-timescale simulations. Regular MD simulations of similar manno-oligosaccharides were reported to fail reproduction of experimental data [150]. Replica-exchange approach implies running periodically swapped parallel replicas of the system at different temperatures. Ensemble of GM9 conformers sampled by this method was consistent with the NMR observables. Populated areas of density maps built for glycosidic linkages of Glc_1_Man_3_ branch of GM9 (Figure 3c) were close to crystallographic conformations of a linear Glc_1_Man_3_ tetrasaccharide (a GM9 determinant recognized by lectins) from PDB.

Force field (or potential energy function) is represented by atomistic parameter set obtained for a considered compound class. Potential energy value can be calculated as a sum of interaction potentials for bonded (covalent bond stretching, angle bending, proper torsions) and non-bonded (electrostatic and van der Waals interactions) terms, and can include other terms (e.g., improper torsions, solvation, hydrogen bonds [151], nonconventional hydrogen bonds [101], for protein-carbohydrate complexes—CH-π stacking interactions [152,153,154,155], CHI Carbohydrate Intrinsic (CHI) energy contribution [156,157]).

**Figure 4:**
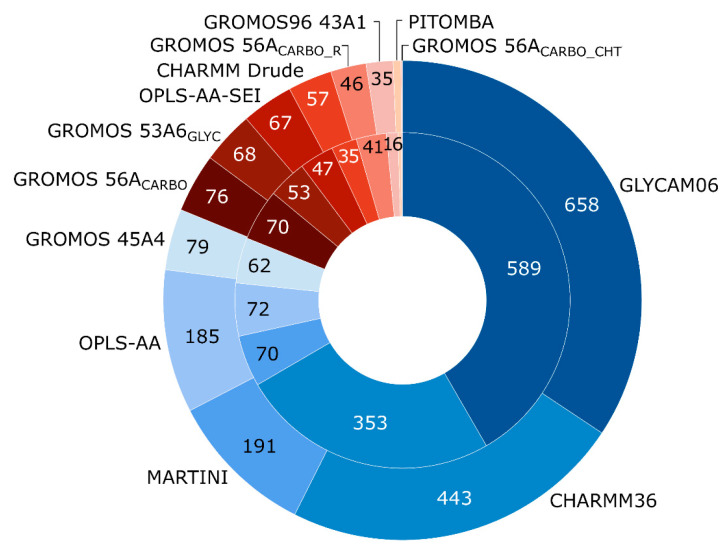
Citations of dedicated force fields in carbohydrate studies for the recent five years, according to Scopus. Outer circle shows total citations (number of citing publications) of force fields in 2015–2020. Inner circle shows citations in articles filtered by a carbohydrate topic. See detailed data, references to original publications, absolute values, and carbohydrate filer details in Supplementary Table S2.

Several force fields developed for general representation of wide range of organic compounds (e.g., Allinger’s MM2, MM3, MM4) can be applied to carbohydrate 3D modeling [151,158,159]. Of them, despite being a universal force field, MM3 [160,161] still exhibits good performance on glycans [162,163,164] (Reviews), [165,166] (exemplary Articles). However, a number of force fields specially tuned for carbohydrates have been developed (Figure 4). In Supplementary Table S2, we provided citation metrics of articles reporting carbohydrate-dedicated and selected general force fields that could be applied to carbohydrate structure modeling. Unfortunately, usage of general force fields could not be adequately estimated via number of citations. Automated full-text analysis and retrieval of data, needed to confirm employment of force fields for carbohydrate molecules, is beyond the scope of this review. Nevertheless, statistical data obtained for general force fields supported in popular MD software packages (e.g., AMBER, CHARMM, GROMACS, Tinker) shows obsolescence of modern force fields above Allinger’s ones, and MM3 in particular (see more detailed data, references to original publications and absolute values in Supplementary Table S2).

**Figure 5:**
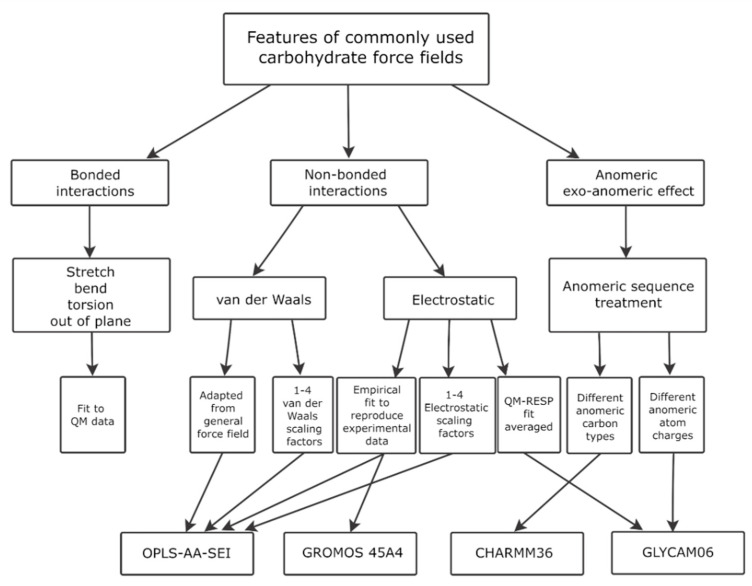
Digest of the most commonly used carbohydrate force fields with parameterization protocol comparison. Reproduced with permission from [138], © 2020 Elsevier Inc.

Detailed comparisons of all-chemical and dedicated force fields in a context of glycan modeling have been published [114,139,151,167]. CHARMM36, GLYCAM06, GROMOS and OPLS-AA-SEI were reported as commonly used force fields for handling carbohydrate or glycoconjugate molecules. More details are provided in Figure 5.

CHARMM36 force field with modern carbohydrate parameter table (C36 [168]) was derived from CHARMM all-atom biomolecular force field [169,170]. Currently, CHARMM36 parameterization features include monosaccharides in furanose [171] and pyranose [172] forms, glycosidic linkages between monosaccharides [171,173], complex carbohydrates and glycoproteins [174], monosaccharide-linked sulfate and phosphate groups [175], acyclic carbohydrates and alditols [171], as well as carbohydrate simulations in aqueous solution [176].

GLYCAM06 force field is compatible with carbohydrates of all ring sizes and conformations for both mono- and oligosaccharides built of residues common for mammalian glycans, such as widespread aldoses, N-acetylated amino-sugars, sialic, glucuronic and galacturonic acids [177]. Parameter set was extended to non-carbohydrate moieties such as lipids [178], glycolipids [179,180], lipopolysaccharides [181], proteins and nucleic acids. Parameterization of GLYCAM06 for glycosaminoglycans was reported [182].

GROMOS represents a broad family of carbohydrate force fields. Having been a classic one since 2005, GROMOS 45A4 [183] parameter set is used for explicit-solvent simulation of hexopyranose-based saccharides. In the recent decade, several parameters of 45A4 were optimized in GROMOS 56A_CARBO_ [184] including lipopolysaccharides [185]. GROMOS 53A6_GLYC_ was improved for explicit-solvent simulations [186] and extended for glycoproteins [187]. GROMOS 56A_CARBO_R_ [188] was designed to improve description of ring conformational equilibria in hexopyranose-based saccharide chains as compared to the previous 56A_CARBO_ version. Another modification of 56A_CARBO_ named 56A_CARBO_CHT_ [189] was developed for chitosan and its derivatives. Recently, extensions of GROMOS 56A_CARBO_/_CARBO_R_ parameter set were adapted towards charged, protonated and esterified urinates [190] and furanose-based carbohydrates [191]. GROMOS96 43A1 was reported to have good performance on glycan structure simulation in glycoproteins [192,193].

OPLS-AA scaling of electrostatic interactions (SEI) force field [194] consists of improved parameters for conformational changes associated with φ-ψ dihedrals combined with enhanced accuracy of QM relative energy calculation in carbohydrate molecules refined for OPLS-AA biomolecular force field [195,196]. Additionally OPLS force field was improved for explicit-water simulations [197].

Rapidly developing CHARMM Drude polarizable force field for carbohydrates based on classical Drude oscillator has to be mentioned. Parameter sets obtained for hexapyranoses [198] and their aqueous solutions [199], aldopentafuranoses and methyl-aldopentafuranosides [200], carboxylate and N-acetylamine saccharide derivatives [201], alditols [202] and glycosidic linkages [203] demonstrated significant improvement of QM data reproduction compared to CHARMM additive force field.

MARTINI coarse-grained (CG) force field [204] can be used alternatively to all-atom (AA) level simulations with advantage of modeling large carbohydrate systems (solutions of oligo-, polysaccharides, glycolipids [205,206,207]) on a long time scale at reasonable computational cost. Blocked ring puckering (only ^4^C_1_ conformation is allowed) and restrictions on the anomeric effect and glycosidic bond flexibility cumulatively provide reduction of available degrees of freedom. Another CG model PITOMBA [208] for carbohydrate simulations was developed based on GROMOS 53A6_GLYC_ force field.

Docking methods for carbohydrate ligands utilize molecular modeling approaches for protein-carbohydrate complexes for initial geometry generation, conformational sampling, grafting, active site mapping and binding affinity estimation [129,137,209,210,211]. Accurate reproduction of experimental data requires application of particular scoring function parameterization (empirical, force fields or knowledge-based [212]) and docking protocols, which depend on the interaction types present in a system (CH-π interactions, CHI-energy, hydrogen bonding, solvent model, influence of solvent molecules inclusion effects, charged moiety etc.) [8,213,214,215,216,217,218,219]. Extension of several docking software packages to handle carbohydrate molecules was reported to improve modeling of biologically relevant systems such as lectin-glycan [220,221], GAG-protein [222,223,224], or antibody-carbohydrate [225].

## 4. Model Building and Analysis Tools

Currently available web-based tools along with standalone software packages were developed to facilitate work with carbohydrate 3D structure. Versatile online services for in silico molecular modeling allow users to start from a user-friendly structure input, and to automatize further procedures (see Table 2 for references). GLYCAM-Web provides tools for glycan structure prediction, glycosylated protein 3D model generation, grafting and docking. CHARMM-GUI modeler offers options for 3D structure generation and modeling of glycans including N-/O-glycoproteins and glycolipids [226,227]. Biological membranes can be simulated with the assistance of CHARMM-GUI Membrane Builder (by combining features of LPS and glycolipid CHARMM-GUI Modelers) and GNOMM (a tool for building lipopolysaccharide-rich membranes). Noteworthy standalone programming frameworks for structure modeling are Glycosylated (modeling of glycans, glycoproteins and glycosylation) and Rosetta Carbohydrate (loop modeling [228], glycan-to-protein docking, and glycosylation modeling).

To build diverse saccharide 3D models online, one can use such tools as REStLESS and SWEET-II. doGlycans standalone framework can be used for preparation of the atomistic models of glycopolymers, glycolipids and glycoproteins. Complex polysaccharide 3D models can be generated via POLYS and CarbBuilder. Another special class of polysaccharide builders is dedicated to glycosaminoglycans (GAGs) which can be accessed using POLYS GAG-builder and GLYCAM-Web GAG-builder. Recently, another approach for building GAG molecules was reported [229] (exemplary data pipeline only). Unfortunately, application scope of the majority of the existing structure building and modeling services is limited to rigidly defined set of supported sugar residues, and lacks non-carbohydrate moiety support.

Tools for locating and identification of a carbohydrate moiety (e.g., pdb2linucs, GlyFinder, Glycan Reader) are useful for the atomic coordinate analysis and extraction of glycoproteins and protein-carbohydrate complexes deposited in Protein Data Bank (PDB). Automated molecular geometry processing facilities can be accessed via glycoinformatics tools designed for conformational data analysis (CAT, BFMP), nuclear Overhauser effect (NOE) calculation (MD2NOE, Distance Mapping) and 3D structural data analysis related to glycan moieties from PDB (GlyTorsion, GlyVicinity, GS-align).

In Table 2, we summarized freely available tools for generation and processing carbohydrate 3D structural data and divided them into eight categories of application.

## 5. Experimental Data Validation

**Figure 6:**
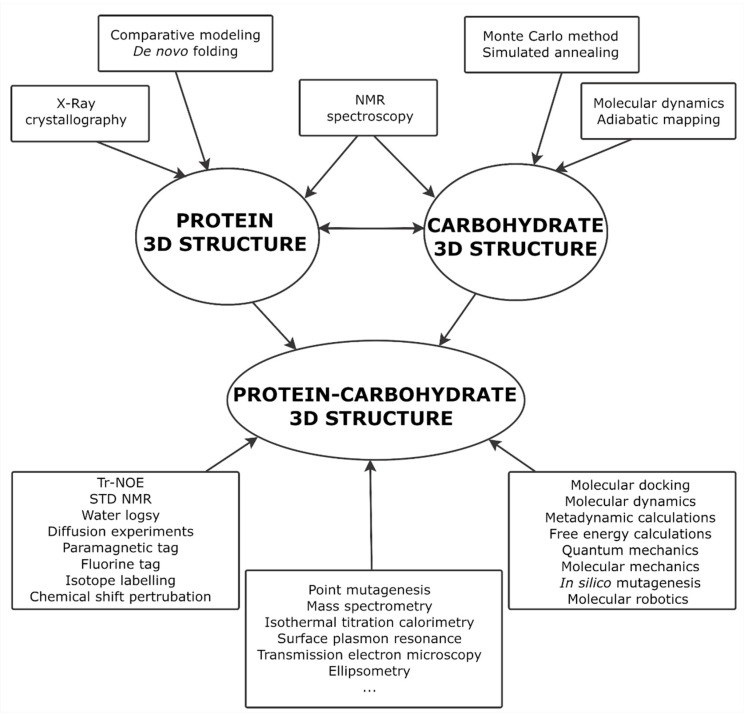
Interplay of the instrumental and computational methods in the 3D structure determination of carbohydrates, proteins, and protein–glycoconjugate complexes. Reproduced from [285] © 2020 The authors. Published by Wiley-VCH Verlag GmbH & Co. KGaA.

Vast variety of methods provide information about 3D structure of individual glycans and glycan moieties of glycoproteins and protein-carbohydrate complexes (Figure 6) [285,286]. The following approaches are most utilized for 3D structural data validation [287,288,289]:

- Ccombination of carbohydrate simulated geometry data with X-ray crystallographic data analysis [225,290];
- Analysis of inter-glycosidic NMR spin couplings, which depend on glycosidic bond torsions [114,291,292];
- Deriving nuclear Overhauser effects (NOEs) from relative populations of the interatomic distances, with subsequent comparison to the experimental NOEs in solution [99,293,294];
- Purely informatic detection of errors, such as incompatible atomic coordinates originating from incorrect processing or simulation [295,296,297,298];
- Simulation by other computational methods at higher levels of theory [102,103,105,108].

Unfortunately, most of the data obtained on the basis of crystallographic experiments can dramatically differ from glycan conformations in solution or have poor resolution which needs further adjustment [299,300]. Moreover, not all of the objects of interest can be obtained as a single crystal. Electron cryo-microscopy gains popularity for carbohydrate 3D structural research [301], however, this method requires additional refinement procedures due to resolution restrictions of the obtained density maps [302,303,304]. Recently, cryo-EM data were used for the refinement of SARS-CoV-2 spike glycoprotein stucture using Privateer (see Table 3 for references) software [305,306].

**Figure 7:**
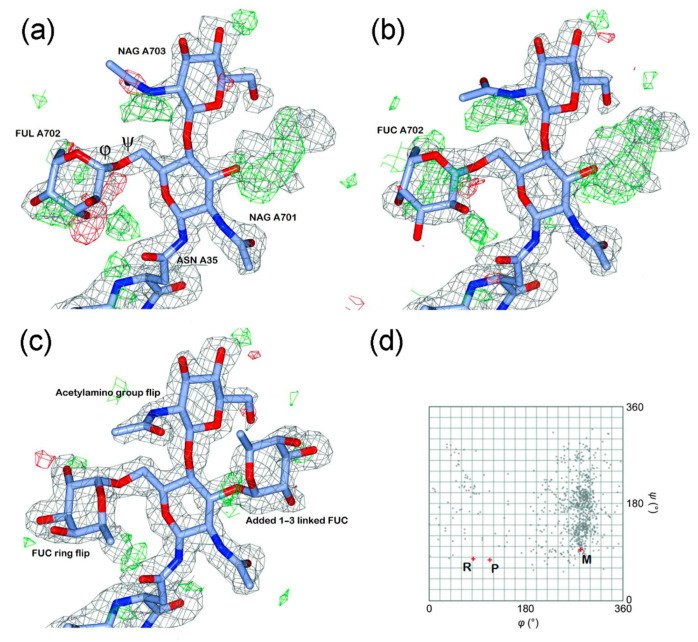
X-ray diffraction data refinement of N-glycan moiety from PDB ID 2Z62. 2mF_o_–DF_c_ electron density map contoured at 1σ is displayed in grey; positive and negative mF_o_–DF_c_ difference electron density maps contoured at 3σ are displayed in green and red, respectively. (**a**) Original glycan structure model from the PDB entry. (**b**) PDB-REDO model with properly renamed fucose residue and improved fit to the electron density. (**c**) Manually rebuilt model based on PDB-REDO results. (**d**) CARP distribution plot for glycosidic φ-ψ torsions of FUC(1-6)NAG (from panel (a)) in PDB. Characteristic points: R, model refined with PDB-REDO; P, original PDB model; M, manually rebuilt model. Reproduced from [295], © 2020 The authors. Published by John Wiley & Sons, Inc.

Van Beusekom et al., illustrated [295] quality improvement of the PDB glycan structure model with incorrect (1–6)-linked fucose annotation, poor fit to the electron density, and missing (1–3)-linked fucose (Figure 7a) with the help of PDB-REDO (Figure 7b) and CARP (Figure 7d) tools (see Table 3 for references). Structure model obtained by PDB-REDO treatment was further manually inspected (Figure 7c): corrections were made for acetylamino group geometry, distorted (1–6)-linked fucose ring conformation, and (1–3)-linked fucose residue was added. Despite successful automated resolution of residue annotation problem and poor electron density refinement, complete revision could not be achieved without manual intervention.

NMR techniques are a powerful approach to investigate conformational and dynamic behavior of carbohydrate moieties in biomolecules [307,308,309,310]. However, the nature of NOE enhancement factor has been hampering obtaining the sufficient number of distance restrains [99]. In the case of saccharides with their multiple rotatable bonds, the stable 3D structure was difficult to define, making molecular modeling essential for this class of compounds. Adjustment of experimental conditions helped to overcome the mentioned limitation and to reproduce crystal structures of oligosaccharides by modeling with NOE-derived distance restraints [100,101].

**Figure 8:**
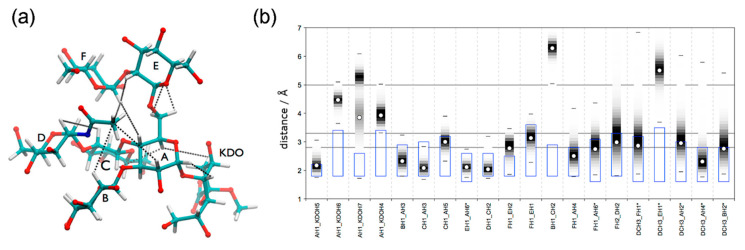
M. catarrhalis lgt2Δ structure validation based on NOE data analysis. (**a**) Characteristic proton-proton contacts; (**b**) NOE-filtered (blue boxes) sampling of proton-proton distances from MD simulation (grey shades). Reproduced from [314], © 2020 The authors. Licensee MDPI, Basel, Switzerland.

Since there is no direct way to derive detailed three-dimensional representation from the observed NOE intensities, additional molecular modeling protocols are required to establish comprehensive view of conformational space at the atomic level [311,312,313]. Frank et al., demonstrated conformation filtering based on the observed NOE obtained by molecular dynamics in explicit solvent [314]. As a representative example, Figure 8 depicts ^1^H-^1^H spatial contacts and conformation selection criteria illustrated by *Moraxella catarrhalis* lgt2Δ bacterium heptasaccharide, which adopts an unusual conformation.

## 6. Protein Data Bank and Its Validation

Protein Data Bank (PDB) [315] and Cambridge Structural Database (CSD) [316] are historically considered the main repositories of experimentally determined carbohydrate three-dimensional structures. CSD is reported to deposit over 4000 crystal structures of oligosaccharides [93]. Unlike Cambridge Structural Database, Protein Data Bank provides open access to the entire structural archive. Carbohydrate moieties deposited in PDB are usually represented as covalently bound to protein or imply non-covalently bound protein-carbohydrate complex formation [302]. According to recent reports, as at November 18, 2019 Protein Data Bank contained ~13500 carbohydrate structures representing ~9.4% of total database records [317].

Despite being a valuable source of 3D structural data for glycoscientists, PDB lacks convenient search facilities for glycan structures. Some projects have developed data-mining tools capable of retrieving bioglycan molecular geometry data from PDB: Glycan Reader (GlycanStructure.org) [260,261] (http://www.glycanstructure.org/), pdb2linucs (GLYCOSCIENCES.de) [47,259,318] (http://www.glycosciences.de/database/start.php?action=form_pdb_data), GlycoNAVI TCarp [61] (https://glyconavi.org/TCarp/) (https://gitlab.com/glyconavi/pdb2glycan) and GlyFinder (GLYCAM-Web) [257,258] (https://dev.glycam.org/portal/gf_home/).

**Figure 9:**
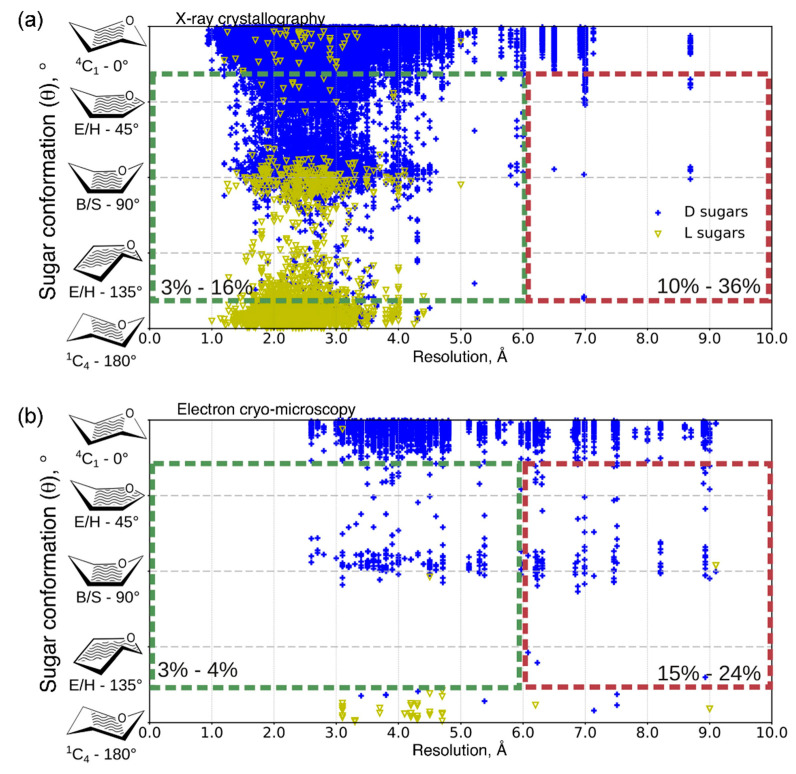
Distribution of D- (shown in blue) and L-pyranoside (shown in yellow) ring conformations as function of resolution for all sugar moieties in N-glycosylated proteins in PDB (on April 2019) solved with (**a**) X-ray crystallography and (**b**) electron cryo-microscopy. Non-chair conformations are bordered by dotted line boxes for 0.0-6.0 Å (green) and 6.0-10.0 Å (red) resolution ranges; the percentage of structures is given in the boxes. Reproduced with permission from [301], © 2020 Elsevier Ltd.

Another issue of concern related to Protein Data Bank is large proportion of errors in deposited coordinates, leading to requirement for a thorough checkup and development of data remediation services [319]. Commonly occurring problems associated with nomenclature, poor glycan geometry, linkage errors, missing or surplus atoms can seriously decline the quality of the obtained 3D structures [300,320,321]. Using Privateer software, it was discovered [299],[301] that PDB deposits significant number of erroneous N-glycosylated structures with pyranose ring distortions, considering preferred adoption of ^4^C_1_ conformation for D-sugars and ^1^C_4_ conformation for L-sugars (Figure 9). In most cases, poor electron density of carbohydrate moiety results in anomalous high-energy pyranose ring conformations (envelopes, half-chairs, boats, skew boats, etc.). To obtain a reasonable structure model, experimental data refinement programs should be applied to derive geometric restraints for sugar monomers. Notably, despite a cryo-EM method has a resolution limit disadvantage, observed results indicate larger content of atypical conformations solved by X-ray crystallography, as compared to cryo-EM data.

Exceptions for the relevancy of high-energy conformations were found in complexes involving carbohydrate-active enzymes, which force pyranose ring distortion enabling catalytic transformation of a carbohydrate substrate via transition states (e.g., glycosydic bond hydrolysis) [322]. Fushinobu has performed glycosidic torsion analysis for a set of PDB entries of crystal structure complexes bound to ligands bearing lacto-N-biose I (LNB, both α- and β-anomers) disaccharide unit presented in type-1 antigens. The study was supported by GlycoMaps DB (see Table 1 for references) [323]. Obtained φ-ψ data for LNBs bound to various proteins was plotted against corresponding free energy maps. Distortion of the energetically favored ring conformation strongly depended on substrate catalytic and recognition mechanisms.

To date, existing tools for carbohydrate structural error detection and correction in PDB files (Table 3) cannot be used directly as an integral part of Protein Data Bank. Nevertheless, initiative aimed at improvement of quality at wwPDB was carried out via collaboration with glycoscience community in July 2020 [324] (https://www.wwpdb.org/documentation/carbohydrate-remediation). It includes data annotation and validation of carbohydrate-containing records.

**Figure 10:**
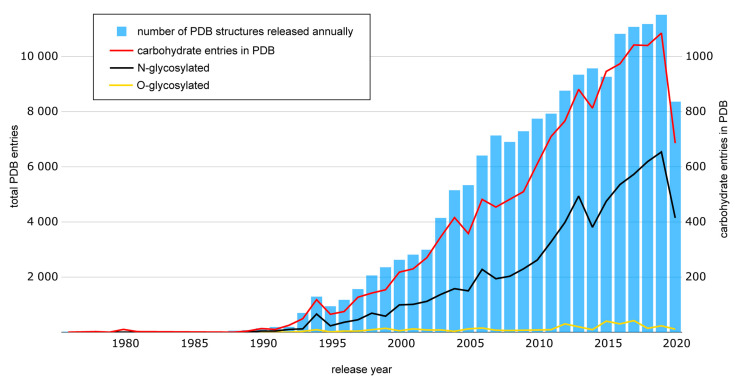
Deposition statistics of carbohydrate-containing structures in Protein Data Bank based on carbohydrate remediated list data. Data for 2020 cover seven of twelve months. See detailed data in Supplementary Tables S3–S4.

Proportion of carbohydrate-containing structures in PDB has been recently reported in [302]. Figure 10 presents our analysis of data published in the framework of Protein Data Bank carbohydrate remediation project. 14117 PDB entries from carbohydrate remediation list (https://cdn.rcsb.org/wwpdb/docs/documentation/carbohydrateRemediation/PDB_carbohydrate_list.list) were sorted by release year and plotted against the growth of PDB structures released annually (https://www.rcsb.org/stats/growth/growth-released-structures) (as on August 10, 2020; 167,327 PDB entries were available). Obtained results indicated that ~8.4% of PDB records contained a carbohydrate moiety. Additionally, each PDBx/mmCIF file corresponding to PDB ID from carbohydrate remediation list was parsed to reveal the presence of N- or O-glycosylation site annotations, which resulted in ~4.2% (7076 N-glycosylated entries) and 0.2% (362 O-glycosylated entries) of total database records. A few S- and C-glycans (24 entries in total) were neglected.

Statistics on glycans in Protein Data Bank was reported [259,302,317,325], as well as tools that could facilitate collection of statistical data (Glycan Reader [70,260,261], GlyFinder [258], pdb2linucs and pdb-care [326]).

## 7. 3D Structure Input and Visualization

Carbohydrate structure visualization in publications and computer interfaces is extremely important in terms of perception universality, unambiguity, and machine-readability. Hence, carbohydrate input [335,336,337] and visualization [338,339] tools are actively developing. Feature comparison of glycan sketchers, builders and viewers (occasionally including 3D ones) was reported in a recently published review [340]. In our review, we gave more emphasis to 3D visualization approaches.

Being informative to represent glycan primary structure, most of graphical input tools such as GlycanBuilder [341], DrawRINGS [342], SugarSketcher [343], DrawGlycan-SNFG [344,345] and GlycoGlyph [337] are inappropriate for obtaining 3D structural models and their visualization due to lack of underlying modeling and insufficient data conversion functionality.

At present, glycan 3D molecular models can be built in user-friendly software allowing constructing glycans from individual saccharide components. Free web-tools, such as GLYCAM-Web, CHARMM-GUI, POLYS glycan builder, GAG-builder, SWEET-II should be noted (more references are listed in Table 2). A few commercial molecular modeling software is equipped with special plugins for glycan 3D structure building based on a list of predefined monosaccharide templates, e.g., Sugar Builder tool in HyperChem (http://www.hyper.com/?tabid=360) software [346] or Azahar [235] plugin in PyMol package (Schrödinger software) (https://pymol.org/2/)[347].

To render 3D glycan structure and its conformational features, it should be recorded using a notation which includes atomic coordinates, such as MOL [348] or PDB [349]. All-atom visualization based on atomic coordinates is supported by the majority of existing molecular modeling software. Several carbohydrate structure databases utilize interactive 3D visualization using open-source software engines. As one of the pioneers, GLYCOSCIENCES.de portal developed PDB2MultiGIF [350] (http://www.glycosciences.de/modeling/pdb2mgif/) visualization pipeline which generates an animated image of 3D model from a PDB file using RasMol [351] (http://www.openrasmol.org/). RasMol visualization was included in W3-SWEET [263] (ancestor of SWEET-II) pipeline developed by same project. Nowadays, more advanced interactive visualization applications have been developed for carbohydrate 3D molecule presentation. Jmol/JSmol [352] (http://www.openrasmol.org/) visualization applet is useful to display 3D models of carbohydrate molecules applied in numerous projects, such as CSDB, GLYCOSCIENCES.de, GLYCAM-Web and EK3D (see references in Table 1). NGL [353,354] (http://nglviewer.org/), LiteMol [355] (https://www.litemol.org/) and Mol* [356] (https://www.rcsb.org/news?year=2020&article=5efe0f606378d876901146f8) (https://molstar.org/) 3D viewers are handy for processing macromolecular PDB data stored in glycoproteomics databases (UniLectin3D, Glycan Binding Site DB, ProCarbDB, GlycoNAVI, ProCaff, etc.; see references in Table 1) and general proteomics repositories such as PDB [315] (http://www.wwpdb.org/), UniProtKB [357] (https://www.uniprot.org/) or SWISS-MODEL [90] (https://swissmodel.expasy.org/repository).

NGL viewer was developed mainly for convenient protein macromolecule structure processing. It allows only ball-stick representation for small molecules or non-peptide fragments, such as saccharide residues. LiteMol (and its successor, Mol*) viewer could be applied for the visualization of an arbitrary glycan with facility of highlighting carbohydrate fragments or displaying specific interactions in protein-carbohydrate complex structure. Due to these features, it was implemented in multiple carbohydrate structure databases (e.g., CSDB, Glyco3D, MatrixDB, and EPS-DB).

Despite the absence of the experimental 3D structural data, a number of carbohydrate databases have opportunity to simulate 3D atomic coordinates for deposited or inputted compounds from primary structure owing to tools developed by glycoinformatics community. CSDB (REStLESS API [265]), GLYCOSCIENCES.de (SWEET-II [264,350]) and GLYCAM-Web (http://glycam.org/) portals make it possible to generate 3D atomic coordinates recorded in PDB (all) and MOL (CSDB) file formats. POLYS developed by Glyco3D project enables the construction of polysaccharides in PDB format; it was introduced in MatrixDB and EPS-DB databases. More details are provided in Table 2.

**Figure 11:**
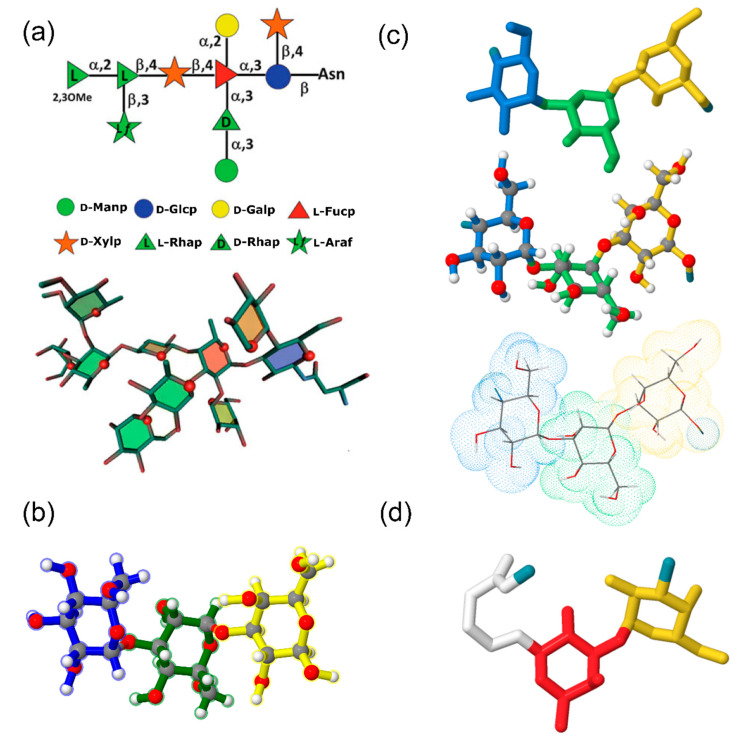
Glycan structure colored according to SNFG, or superimposed with 3D SNFG, as implemented in SweetUnityMol (**a**), GLYCOSCIENCES.de (via JSmol) (**b**), and CSDB (via JSmol) (**c**,**d**), see text. Panel (a) was reproduced with permission from [372], © Springer Japan 2017.

**Figure 12:**
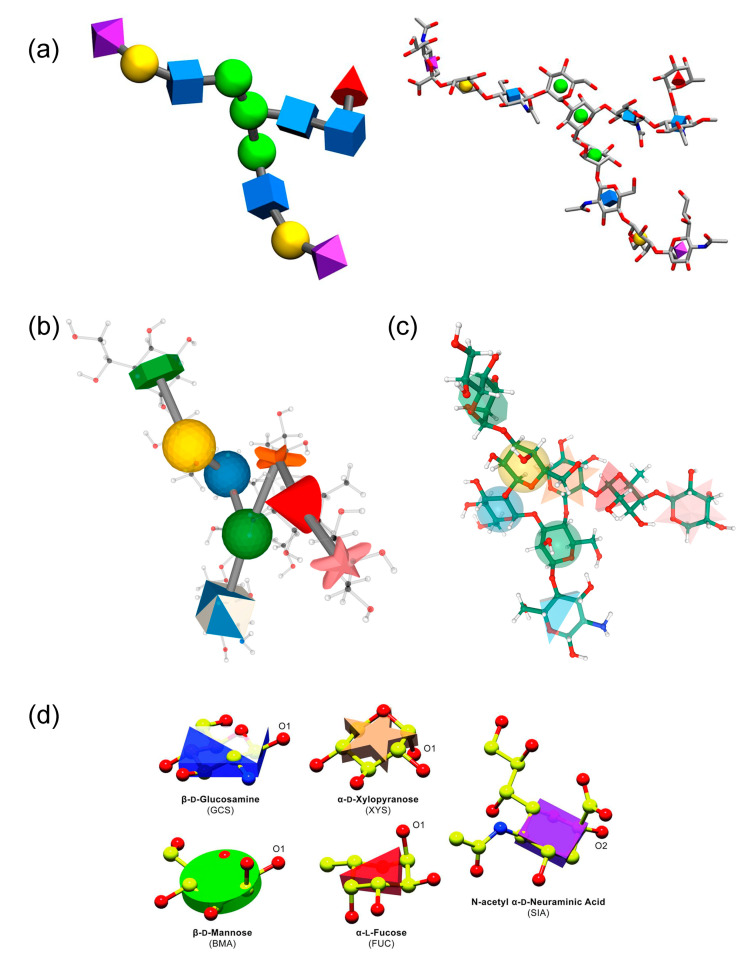
Glycan structure colored according to SNFG, or superimposed with 3D SNFG, as implemented in 3D-SNFG (**a**), LiteMol (**b**), Mol* (**c**); monosaccharide presentation in Glycoblocks (**d**). Panel (a) was reproduced with permission from [366], © 2020, Oxford University Press. Panel (d) was reproduced from [369], © 2020 The authors. Published by John Wiley & Sons, Inc.

Atomic coordinates and all-atom molecular models have not been popular in publications due to a lack of human readability. First attempts [358,359] of prof. Kuttel et al., to visualize carbohydrate molecules in an efficient and simple way were made by developing PaperChain and Twister graphic algorithms as a part of CarboHydra [360] and Visual Molecular Dynamics [361] software packages. Later, group of prof. Pé rez suggested to restrict visualized molecule to skeletal atoms via conditional cycle plane coloring in accordance with the color code adopted in SNFG [338] visualization scheme (SweetUnityMol software [362], Figure 11a). Another UnityMol visualization approach called Umbrella Visualization [363,364] was tailored for N-glycan structures. Azahar plugin for PyMol [235] affords cartoon models with polygons and rods. Several solutions for convenient visualization came up with the development of SNFG notation [339]. Thus, group of prof. Woods proposed to combine molecular structure elements with 3D SNFG icons (Figure 12a). Such convenient visualization technique was integrated in LiteMol (Figure 12b) [365] and Mol* (Figure 12c) [324,356]. 3D SNFG visualization plugins are available via Visual Molecular Dynamics platform [366] (http://glycam.org/docs/othertoolsservice/2016/06/03/3d-symbol-nomenclature-for-glycans-3d-snfg/) and UCSF Chimera [367] visualization software Tangram plugin (https://github.com/insilichem/tangram_snfg). Designed as part of CCP4mg [368] molecular-graphics software, Glycoblocks [369] representation of monosacchrides uses shapes and colors, identical to those in SNFG (Figure 12d). Available as PyMol plugin developed by Widmalm group (http://www.organ.su.se/gw/doku.php?id=3dcfg), 3D-CFG representation [370] based on CFG notation [371] (often referred to as a predecessor of SNFG) should also be noted as earlier approach to interpretation of carbohydrate 3D structures based on a symbol library. 

Considering efficiency and usability of 3D representation based on SNFG concept, which grows popular among glycoscientists, the development of alternative solutions in carbohydrate 3D structure representations has a potential for application in glycoinformatics projects. Support of colored residues in 3D structures implemented via JSmol on GLYCOSCIENCES.de portal was reported [47] (Figure 11b). Similarly, CSDB project has developed a 3D viewer (http://csdb.glycoscience.ru/database/core/show_3d.php?csdb=-3)aDManp(1-3)[Ac(1-2)?DGlcpN(1-6)]bDGal?(1-) with carbohydrate residue coloring according to the SNFG notation in the framework of a modeling module based on REStLESS API. In this tool, user can visualize input structure with help of sticks, balls and sticks, or van der Waals spheres (Figure 11c). Options for aglycone moiety (white) and pseudo-atoms (polymeric repeats, blue caps) are supported (Figure 11d).

## 8. Conclusions

Development of glycoinformatics resources makes great impact on treating enormous masses of data sets produced by glyco-related research. Tools for carbohydrate 3D structural information retrieval provide a framework for experimental and computational data quality validation. Data sources based on conformational ensemble generation and analysis assist structure–function and structure–activity relationship prediction of biologically relevant bioglycans and glycoconjugates. In this review, we have summarized existing facilities on working with glycan spatial features that can provide harmonious network of structural databases, web-services, tools and standalone software for modeling and processing structural data. Further advances in this field will help building better understanding of glycan participation in biological processes and supply glycoscience community with user-friendly access to voluminous data collections.

## Figures and Tables

**Table 1:** Carbohydrate databases with 3D structure support.

Database	Years ^a^	Description ^b^	Data Coverage	Carbohydrate 3D Structures	References
*Structure-centric*					
Carbohydrate Structure Database (CSDB) 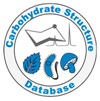	2005– present	structures from prokaryotes, plants, and fungi taxonomy diseases bibliography *curated*	24669 structures 12521 organisms 9353 publications 2096 glycosyltransferase activities 13378 NMR spectra (^1^H, ^13^C)	1327 disaccharide conformational maps 3D atomic coordinate generation	[28,42,43,44] (http://csdb.glycoscience.ru/database)
Glycosciences.DE 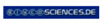	1997– present	taxonomy bibliography	26559 structures 20211 publications 3434 NMR spectra (^1^H, ^13^C)	13599 3D structure models 12098 PDB entries (1880 distinct glycan structures) 2585 conformational maps 3D atomic coordinate generation	[45,46,47] (http://www.glycosciences.de/)
Glyco3D 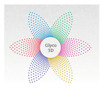	2015– present	taxonomy bibliography *curated*	245 monosaccharides 125 disaccharides 314 bio-oligosaccharides 140 polysaccharides 415 GT structures 88 mAb structures 46 GAG structures 1662 lectin structures X-ray data * NMR data * molecular modeling data *	3035 3D structures * PDB entries * disaccharide conformational maps * 3D atomic coordinate generation	[48,49] (http://glyco3d.cermav.cnrs.fr/home.php)
PolySac3DB 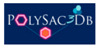	2012– present	polysaccharides taxonomy bibliography *curated*	157 structures 84 publications X-ray data * NMR data * molecular modeling data *	157 3D structures PDB entries * conformational maps *	[50] (http://glyco3d.cermav.cnrs.fr/home.php)
EK3D 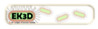	2016– present	*E. coli* K antigens bibliography *curated*	molecular modeling data protein data	72 3D structures 3D atomic coordinate generation	[51] (www.iith.ac.in/EK3D/)
3DSDSCAR 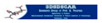	2010– present	sialic acid-containing oligosaccharides aqueous molecular dynamics simulations	27 structures	92 3D conformational models	[52,53] (http://aliffishbay.com/Domains/3dsdscar.org/3dsdscar.html)
MatrixDB 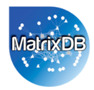	2011– present	protein–polysaccharide interactions taxonomy genetic data bibliography *curated*	58 GAG sequences proteoglycan structures * 1507 experiments 1058 experimentally supported associations 269 publications	3D structures * PDB entries * 3D-atomic coordinates generation (GAGs)	[54,55,56] (http://matrixdb.univ-lyon1.fr/)
EPS-DB 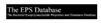	2017– present	bacterial exopolysaccharides functional properties genetic data taxonomy bibliography *curated*	105 structures	85 3D structure models 3D-atomic coordinates generation	[57] (http://www.epsdatabase.com)
GlyMDB 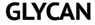	2020– present	glycan microarrays	5203 glycan microarray samples	1965 3D structures (PDB entries) 771 3D structures with glycan ligands (PDB entries)	[58] (http://www.glycanstructure.org/glymdb/)
CFG Glycan Structures Database 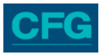	2006– present	mammalian glycan arrays taxonomy biological sources diseases bibliography	N-glycans * O-glycans *	3D-atomic coordinates generation	[59,60] (http://www.functionalglycomics.org/glycomics/molecule/jsp/carbohydrate/carbMoleculeHome.jsp) (http://www.functionalglycomics.org/glycomics/publicdata/selectedScreens.jsp)
*Glycoproteomic*					
GlycoNAVI Tcarp 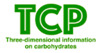	2020– present	diseases genetic data taxonomy bibliography	2723 unique analyzed glycans 5814 glycoproteins 712 lectins	3D structures * 15003 PDB entries 3D atomic coordinate generation	[61] (https://glyconavi.org/TCarp/)
GlyCosmos 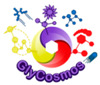	2017– present	diseases genetic data taxonomy	109854 glycans glycolipids * 50113 glycoproteins 1238 lectins 20580 glycogenes	3D structures (PDB and UniProtKB entries) *	[13,62,63] (https://glycosmos.org/)
SugarBind 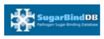	2010– present	adherence to pathogens taxonomy diseases bibliography *curated*	739 lectins 204 glycan ligands 567 pathogenic agents 1266 bindings 183 publications	3D lectin structures (PDB entries) *	[64] (https://sugarbind.expasy.org/)
GlyConnect 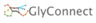	2019– present	protein glycosylation taxonomy biological sources diseases bibliography *curated*	2662 glycoproteins 3609 glycans 246 organisms 5675 sites 913 publications	3D glycoprotein structures (PDB entries) *	[65] (https://glyconnect.expasy.org/)
ProGlycProt 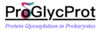	2012– present	prokaryotes taxonomy bibliography homology models * *curated*	crystal structures 61 glycoproteins 62 glycosyltransferases 38 enzymes/proteins involved in protein glycosylation 518 publications	3D structures (PDB entries) * 3D homology models (UniProtKB entries) *	[66,67] (http://www.proglycprot.org/)
ProCarbDB 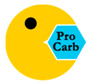	2020– present	protein-carbohydrate complexes taxonomy bibliography binding affinities *curated*	5254 complexes 867 ligand monomers X-ray data	5254 3D structures (PDB entries)	[68] (http://www.procarbdb.science/procarb/)
Procaff 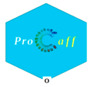	2019– present	protein-carbohydrate complexes taxonomy bibliography	3122 entries 228 publications 125 organisms 354 proteins 835 carbohydrates thermodynamic data	335 3D structures (PDB entries)	[69] (https://web.iitm.ac.in/bioinfo2/procaff/index.html)
GBSDB 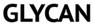	2020– present	protein-carbohydrate complexes *curated*	6402 carbohydrate-containing PDB structures 12075 binding sites	6402 3D structures (PDB entries)	[70] (http://www.glycanstructure.org/gbs-db/pdb/)
PROCARB 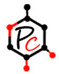	2010– present	protein-carbohydrate complexes	604 complexes 48 modeled glycoproteins 100 unique carbohydrate ligands	604 complexes 3D structures (PDB entries) 26 N-linked 3D homology models 22 O-linked 3D homology models	[71] (http://www.procarb.org/procarbdb/)
UniLectin3D	2019– present	lectins taxonomy bibliography *curated*	2207 structures (1401 interacting with glycan) 535 distinct lectin sequences 228 distinct glycans 896 publications X-ray data	3D structures (PDB entries) *	[72,73] (https://www.unilectin.eu/unilectin3D/)
Lectin Frontier 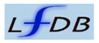	2015– present	lectins taxonomy bibliography	398 structures binding affinities	3D structures (PDB entries) *	[74] (https://acgg.asia/lfdb2/)
LectinDB 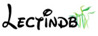	2006– present	lectins taxonomy (all domains, incl. viruses) bibliography *curated*	789 organisms 821 PDB entries	PDB entries *	[75] (http://proline.physics.iisc.ernet.in/lectindb/)
GlycoEpitope 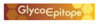	2006– present	epitopes taxonomy diseases functions receptors bibliography *curated*	178 epitopes 624 antibodies	PDB entries (epitopes) *	[76,77,78] (https://www.glycoepitope.jp/epitopes)
GlycoCD	2012– present	glycan CD antigens bibliography *curated*	19 glycan CDs 44 CRD-CDs	PDB entries *	[79] (http://www.glycosciences.de/glyco-cd/)
SACS	2002– present	antibodies automatically-updated	3994 entries crystal/EM structure data	PDB entries *	[80] (http://www.bioinf.org.uk/abs/sacs/xslt.cgi?src=antibodies.xml&xsl=summary.xsl)
SabDab 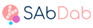	2014– present	antibodies automatically-updated taxonomy binding affinities *curated*	4223 entries 111 carbohydrate-containing antigen types experimental data	111 3D structures (PDB entries)	[81] (http://opig.stats.ox.ac.uk/webapps/newsabdab/sabdab/)
CAZy 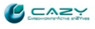	1998– present	carbohydrate-active enzymes and carbohydrate-binding modules taxonomy genetic data bibliography *curated*	CAZy structures * CAZy activities *	7500 ^c^ 3D structures bearing glycan-containing ligand or a glycan analog revealing enzyme-glycan interactions (PDB entries)	[82,83,84] (http://www.cazy.org/)
dbPTM 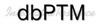	2006– present	protein post-translational modifications taxonomy diseases genetic data bibliography *curated*	32 C-linked glycosylations 3289 N-linked glycosylations 1860 O-linked glycosylations 6 S-linked glycosylations	3D structures (UniProtKB entries) *	[85,86,87] (http://dbptm.mbc.nctu.edu.tw/)
SWISS-MODEL Repository 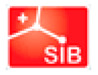	2004– present	3D protein homology models taxonomy regularly updated	glycoprotein structures * 1698194 models from SWISS-MODEL for UniProtKB 158670 structures from PDB with mapping to UniProtKB	3D structures (PDB and UniProtKB entries) *	[88,89,90] (https://swissmodel.expasy.org/repository)
*Specialized*					
GlycoMaps DB 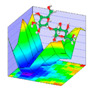	2004– present	di- to pentasaccharides	*in vacuo* high-temperature molecular dynamics	conformational maps for 2585 glycosidic linkages	[91] (http://www.glycosciences.de/modeling/glycomapsdb/)
GFDB 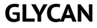	2013– present	glycosidic torsion angles clustering analysis	1754 ^c^ unique glycan sequences in PDB 9055 ^c^ unique fragments with chemical modifications 127202 ^c^ fragment structures	PDB entries * 3D-atomic coordinates generation	[92] (http://www.glycanstructure.org/fragment-db)
GLYCAM-Web 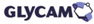	2013– present	mammalian glycans	pre-built libraries of predicted 3D structures of common bioglycans	3D structure models * 3D-atomic coordinates generation	(http://glycam.org/Pre-builtLibraries.jsp)

^a^ Where unknown, the year of the first publication is given. ^b^ Database is marked as *curated* if manual verification of data was reported in the original publication or at the database web site. ^c^ Published coverage data can be outdated; database interface provides no statistics on current coverage. * Database provides no search facilities for indicated carbohydrate 3D structural data.

**Table 2:** Informatics tools for carbohydrate and glycoprotein modeling, 3D structure prediction and analysis.

Tool	Description	Type ^a^	Reference
*Structure modeling*			
CHARMM-GUI Glycan Modeler	*In silico* N-/O-glycosylation of proteins;modeling of carbohydrate-only systems	Web-service	[230] (http://www.charmm-gui.org/?doc=input/glycan)
CHARMM-GUI Glycolipid/LPS Modeler	Glycolipid and lipoglycan structure modeling	Web-service	[230] (http://charmm-gui.org/?doc=input/glycolipid) (http://charmm-gui.org/?doc=input/lps)
Glycosylator	Rapid modeling of glycans and glycoproteins (including glycosylation) based on CHARMM force field	Python framework	[231] (https://github.com/tlemmin/glycosylator)
RosettaCarbohydrate	Modeling a wide variety of saccharide and glycoconjugate structures (including loop modeling, glyco-ligand docking and glycosylation)	Python framework	[228,232,233,234] (https://www.rosettacommons.org/docs/latest/application_documentation/carbohydrates/WorkingWithGlycans)
Azahar	Monte Carlo conformational search and trajectory analysis of glycans	Python framework; PyMol plugin	[235] (https://github.com/BIOS-IMASL/Azahar)
Shape	Carbohydrate-dedicated fully automated MM3-based conformation simulation	Standalone software	[236] (https://sourceforge.net/projects/shapega/)
Glydict	MM3-based N-glycan structure prediction based on MD simulations	Web-service	[237] (http://www.glycosciences.de/modeling/glydict/)
GLYGAL	MM3-based conformational analysis of oligosaccharides	Standalone software	[238]
Fast Sugar Structure Prediction Software (FSPS)	Automatic structure prediction tool for oligo- and polysaccharides in solution	Standalone software	[239,240,241,242]
*Glycosylation modeling and grafting*			
GLYCAM-Web Glycoprotein Builder	Attaching a glycan (user input) to a protein (PDB file)	Web-service	(http://glycam.org/gp)
GlyProt	*In silico* generation of N-glycosylated 3D models of proteins	Web-service	[243] (http://www.glycosciences.de/modeling/glyprot/php/main.php)
Phenix CarboLoad	Loading a carbohydrate structure into protein model and PDB file generation	Python framework	[244] (https://www.phenix-online.org/documentation/reference/carbo_load.html)
GLYCAM-Web GlySpec (Grafting)	Prediction of glycan specificity by integrating glycan array screening data and 3D structure	Web-service	[245,246,247,248,249] (http://glycam.org/djdev/grafting/)
*Biological membranes and micelles*			
CHARMM-GUI Membrane Builder	Building complex glycolipid-/LPS-/LOS-containing biological membrane systems	Web-service	[230,250,251,252,253] (http://www.charmm-gui.org/?doc=input/membrane.bilayer)
GNOMM (gram-negative outer membrane modeler)	Automated building of lipopolysaccharide-rich bacterial outer membranes (3D model preparation for MD simulations in GROMACS)	Standalone software	[254] (http://thalis.biol.uoa.gr/GNOMM/)
Micelle Maker	Micelle building based on broad range of starting lipids and glycolipids (3D model preparation using AMBER software package and GLYCAM library)	Web-service	[255] (http://micelle.icm.uu.se/)
*Carbohydrate moiety identification*			
Cheminformatics Tool for Probabilistic Identification of Carbohydrates (CTPIC)	Identification of small saccharides and their derivatives (input in SDF or MOL format)	Web-service	[256] (http://ctpic.nmrfam.wisc.edu/) (https://github.com/htdashti/ctpic)
Sails	Automated identification of linked sugars	Python framework	(https://github.com/glycojones/sails)
GlyFinder	Locating relevant carbohydrate-containing structures in Protein Data Bank	Part of web-service pipeline	[257,258] (https://dev.glycam.org/portal/gf_home/)
pdb2linucs	Extraction of carbohydrate data from a PDB file	Web-tool	[259] (http://www.glycosciences.de/tools/pdb2linucs/)
GLYCAM-Web PDB-preprocessor	Processing of PDB files with (glyco-)proteins for AMBER-style output	Web-service	(http://glycam.org/pdb)
Sugar identification program	Identifying the residue names of carbohydrates in a PDB file	Standalone software	(http://glycam.org/docs/othertoolsservice/downloads/downloads-software/)
Glycan Reader	Automated sugar identification and simulation preparation for carbohydrates and glycoproteins in PDB files	Web-service	[260,261] (http://glycanstructure.org/glycanreader/) (http://www.charmm-gui.org/?doc=input/glycan)
*Structure building and model preparation*			
doGlycans	Preparing carbohydrate structures (including polysaccharides, glycolipids and glycoproteins) for GROMACS atomistic simulations	Python framework	[262] (https://bitbucket.org/biophys-uh/doglycans/src/master/)
GLYCAM-Web Carbohydrate builder	3D structure prediction of carbohydrates and related macromolecules using GLYCAM06 force field and MD in AMBER (successor of GLYCAM Biomolecule Builder (http://glycam.org/old/biombuilder/biomb_index.jsp))	Web-service	[177] (http://glycam.org/)
SWEET-II	Rapid 3D model construction of oligo- and polysaccharides with MM3 optimization	Web-service	[263,264] (http://www.glycosciences.de/modeling/sweet2/)
REStLESS API	3D structure generation of carbohydrates and derivatives from CSDB Linear notation with MMFF94 optimization (including aglycone moiety)	Web-service	[265] (http://csdb.glycoscience.ru/database/core/translate.html#from)
*Polysaccharide builders*			
POLYS	3D structure generation of poly- and complex oligosaccharides from MM2-precalculated glycosidic linkage torsions and energy minimization	Web-service	[266,267] (https://bitbucket.org/polys/polys/src/default/) (http://glycan-builder.cermav.cnrs.fr/)
CarbBuilder	Building of 3D structures of polysaccharides in CHARMM force field from pre-calculated glycosidic linkage torsions	Standalone software	[268,269] (https://people.cs.uct.ac.za/~mkuttel/Downloads.html)
GAG-builder	Translating of GAG sequences into 3D models based on POLYS glycan builder	Web-service	[270] (http://glycan-builder.cermav.cnrs.fr/gag/) (http://matrixdb.univ-lyon1.fr/)
GLYCAM-Web GAG Builder	Modeling of GAG 3D structure in GLYCAM06 force field using AMBER MD package	Web-service	[271] (http://glycam.org/gag)
*Docking*			
BALLDock/SLICK	Protein-carbohydrate complex docking software	Standalone software, a module in docking software	[272,273] (https://ball-project.org/download/)
HADDOCK	Modeling of biomolecular complexes with support of glycosylated proteins	Web-service	[274] (https://wenmr.science.uu.nl/haddock2.4/library)
Vina-Carb	CHI-energy functions implemented in AutoDock Vina software	Standalone software	[156,157] (http://glycam.org/docs/othertoolsservice/download-docs/publication-materials/vina-carb/)
GLYCAM-Web Antibody docking	Docking of an antibody (from a PDB file) to a glycan antigen (from a library or user input)	Web- service	(http://glycam.org/ad)
Cluspro	Sulfated GAG docking (as one of options)	Web-service	[275,276] (https://cluspro.bu.edu/login.php)
GAGDock (DarwinDock)	Modification of DarwinDock method for sulfated glycosaminoglycans	Algorithm	[277]
GlycoTorch Vina	Docking of sulfated glycosaminoglycans based on Vina-Carb	Standalone software	[278] (http://ericboittier.pythonanywhere.com/)
*Structural data analysis*			
Conformational Analysis Tool (CAT)	Analysis of carbohydrate molecular trajectory data derived from MD simulations	Standalone software	[279] (http://www.md-simulations.de/CAT/)
Best-fit, Four-Membered Plane (BFMP)	Analysis of conformational data from crystal structures and MD simulations of carbohydrates	Standalone software	[280] (http://glycam.org/docs/othertoolsservice/download-docs/publication-materials/bfmp/)
Distance Mapping	Estimation of nuclear Overhauser effects in disaccharides	Web-tool	(http://www.glycosciences.de/modeling/distmap/)
MD2NOE	Calculation of Nuclear Overhauser effect build-up curves from long MD trajectories	Standalone software	[281] (http://glycam.org/docs/othertoolsservice/download-docs/publication-materials/md2noe/)
GS-align	Glycan structure alignment and similarity calculation	Standalone software	[282] (http://www.glycanstructure.org/gsalign)
GlyTorsion	Analysis of torsion angles in carbohydrates from Protein Data Bank	Web-tool	[283] (http://www.glycosciences.de/tools/glytorsion/)
GlyVicinity	Analysis of amino acids in the vicinity of carbohydrate residues derived from Protein Data Bank	Web-tool	[284] (http://www.glycosciences.de/tools/glyvicinity/)

^a^ Web-service implies an automated pipeline for running a specific software (e.g., molecular modeling, structure building, carbohydrate coordinate extraction, format conversion). It results in 3D structural data output starting from primary structure input or atomic coordinate file upload. Web-tool is employed for 3D structural data processing and analysis without 3D structural data output; it is a simpler application designed primarily for statistics and visualization. Other types are self-explanatory.

**Table 3:** Tools for structural validation of carbohydrates.

Tool	Description	Type ^a^	Reference
CNS	Macromolecular structure determination and refinement (including carbohydrates and glycoproteins) based on X-ray and NMR data	Standalone software	[327,328,329,330] (http://cns-online.org/v1.3/)
pdb-care	Identification and assigning carbohydrate structures using atom types and coordinates from PDB files	Web-tool	[326] (http://www.glycosciences.de/tools/pdb-care/)
CARP	Glycoprotein 3D quality evaluation based on the analysis of glycosidic torsion angles from PDB	Web-tool	[283] (http://www.glycosciences.de/tools/carp/)
GlyProbity	Accuracy and internal consistency check of carbohydrate 3D structures	Part of web-service pipeline	[257] (https://dev.glycam.org/portal/gf_home/)
PDB2Glycan	3D structure analysis and validation of glycoprotein PDB entries	Part of web-service pipeline	[61] (https://glyconavi.org/TCarp/) (https://gitlab.com/glyconavi/pdb2glycan)
PDB-REDO	Glycoprotein structure model improvement and validation	Web-service; standalone software	[295,325] (https://pdb-redo.eu/)
Coot	Refinement and validation of glycoprotein 3D structure from cryoEM and X-ray crystallography data	Standalone software	[298,331] (https://www2.mrc-lmb.cam.ac.uk/personal/pemsley/coot/)
Rosetta Carbohydrate	Refinement of glycoprotein 3D structure from cryoEM and X-ray crystallography data, based on correction of conformational and configurational errors in carbohydrates	Python framework	[296] (https://www.rosettacommons.org/docs/latest/application_documentation/carbohydrates/WorkingWithGlycans)
Privateer	Automated validation of carbohydrate conformation data based on 3D structure analysis	Standalone software	[297,332] (https://smb.slac.stanford.edu/facilities/software/ccp4/html/privateer.html)
Phenix	Determination, refinement and validation of macromolecular structure (including carbohydrates and glycoproteins) from cryoEM, X-ray diffraction and neutron diffraction crystallography data	Standalone software	[244] (http://phenix-online.org/)
Motive Validator	Automatic custom residue validation in biomolecules, including carbohydrates	Web-service	[333] (https://webchem.ncbr.muni.cz/Platform/MotiveValidator)
ValidatorDB	Pre-computed validation results of ligands and non-standard residues in PDB (including carbohydrates)	Web-service	[334] (http://webchem.ncbr.muni.cz/Platform/ValidatorDb)

^a^ See footnote ^a^ to Table 2.

## Supplementary Materials

Supplementary Materials can be found at https://www.mdpi.com/1422-0067/21/20/7702/s1. Supplementary Tables: S1. Application rate for quantum mechanics and molecular mechanics methods in 2015–2020. S2. Citing of force fields in 2015-2020. S3. Protein Data Bank statistics. S4. PDB carbohydrate remediation list data.

## Abbreviations

3D	Three-dimensional
AA	All-atom
CAZy	Carbohydrate-Active Enzyme
CD	Cluster of Differentiation
CFG	Consortium for Functional Glycomics
CG	Coarse-grained
CHI	Carbohydrate Intrinsic
CRD	Carbohydrate Recognition Site
Cryo-EM	Electron cryo-microscopy
CSD	Cambridge Structural Database
DFT	Density Functional Theory
FUC	α-L-fucopyranose
GAG	Glycosaminoglycan
GAMD	Gaussian Accelerated MD
GBP	Glycan-Binding Protein
GM9	Glc_1_Man_9_GlcNAc_2_
HPLC	High Performance Liquid Chromatography
HREX	Hamiltonian Replica-Exchange MD
INIOM	Our own N-layered integrated molecular orbital and molecular mechanics
LNB	Lacto-N-biose I
MD	Molecular Dynamics
MM	Molecular Mechanics
MS	Mass-spectrometry
msesMD	Multidimensional swarm-enhanced sampling MD
NAG	2-acetamido-2-deoxy-β-D-glucopyranose
NMR	Nuclear Magnetic Resonance
NOE	Nuclear Overhauser Effect
PDB	Protein Data Bank
PDBe	Protein Data Bank Europe
PDBj	Protein Data Bank Japan
PDBsum	Database of Structural Summaries of PDB Entries
QM	Quantum Mechanics
RCSB PDB	Research Collaboratory for Structural Bioinformatics Protein Data Bank
REMD	Replica-exchange MD
SNFG	Symbol Nomenclature for Glycans
UniProtKB	UniProt Knowledgebase
wwPDB	Worldwide Protein Data Bank
